# Aberrant Methylation of MEG3 Functions as a Potential Plasma-Based Biomarker for Cervical Cancer

**DOI:** 10.1038/s41598-017-06502-7

**Published:** 2017-07-24

**Authors:** Jun Zhang, Tingting Yao, Zhongqiu Lin, Yali Gao

**Affiliations:** 1 Department of Obstetrics and Gynecology, The Second Clinical Medical College (Shenzhen People’s Hospital), Jinan University, Shenzhen, 518020 People’s Republic of China; 2 Department of Gynecological Oncology, Sun Yat-sen Memorial Hospital, Sun Yat-sen University, Guangzhou, 510120 People’s Republic of China; 3 Department of Ophthalmology, The Second Clinical Medical College (Shenzhen People’s Hospital), Jinan University, Shenzhen, 518020 People’s Republic of China

## Abstract

Methylation alterations of specific genes have recently been identified as diagnostic biomarkers for human cancers. Although MEG3 has been proved to be a tumor suppressor in cervical cancer according to our previous study, the diagnostic value of MEG3 methylation in plasma is still unknown. Therefore, the aim of this study is to identify a novel epigenetic biomarker for cervical cancer. In the current study, the level of MEG3 methylation was evaluated using methylation-specific polymerase chain reaction. The results showed that the level of MEG3 methylation was significantly higher in cervical cancer tissues and patients’ plasmas than those in adjacent normal tissues and plasmas of healthy participants respectively. Moreover, the accuracy was good enough for MEG3 methylation in plasma to discriminate CIN III patients from healthy participants. In addition, MEG3 methylation in plasma also has high discriminating power to predict HR-HPV infection and lymph node metastasis. Furthermore, hypermethylation of MEG3 in plasma was associated with worse recurrence-free and overall survival in cervical cancer patients. In conclusions, MEG3 methylation in plasma can serve as a diagnostic and prognostic biomarker for cervical cancer, providing useful information for clinical management.

## Introduction

Cervical cancer is the leading cancer which originated from the female reproductive system^1^. In 2011, new cases of cervical cancer were estimated to be about 490,000 worldwide, and deaths caused by this disease would be more than 275,000 which most occurred in developing countries^2^. Despite pap smear screening and other diagnostic techniques are used widely, the overall survival of cervical cancer patients remains poor^3^. Therefore, further understanding the mechanisms of tumorigenesis and development which contribute to prevention and cure of cervical cancer is urgent.

Long non-coding RNA (lncRNA) is RNA molecule that is more than 200 nt in length with limited or no protein-coding capacity^4^. LncRNAs are known to participate in a wide range of biological processes, including the modulation of apoptosis, proliferation, invasion and so on^5–7^. Human maternally expressed gene 3 (MEG3) is a maternally expressed imprinted gene that encodes a length of 1.6 kb nucleotides which suggests to function as a non-coding RNA^8^. Recent studies have demonstrated that MEG3 is abnormal expressed in various human cancers, such as retinoblastoma^9^, colorectal cancer^10^, and hepatocellular carcinoma^11^. More important, our previous study has proved that MEG3 was down-regulated and affected cell proliferation and apoptosis in cervical cancer^12^. However, little is known about the diagnostic value of MEG3, especially MEG3 methylation status, as a plasma-based biomarker for cervical cancer patients.

In present study, we mainly focused on the diagnostic value of MEG3 methylation in plasma base on our previous study. We first confirmed the reliability of MEG3 methylation in plasma of cervical cancer patients. Then we found that MEG3 methylation in plasma was an effective biomarker for the diagnosis of CIN III (cervical intraepithelial neoplasia), HR-HPV (High risk-Human papillomavirus) infection and lymph node metastasis. Lastly, we demonstrated that MEG3 methylation in plasma was a prognostic factor for cervical cancer patients. Together, these results were to identify the clinical significance of plasma MEG3 methylation in cervical cancer.

## Results

### Reliability of MEG3 methylation in plasma

**Figure 1:**
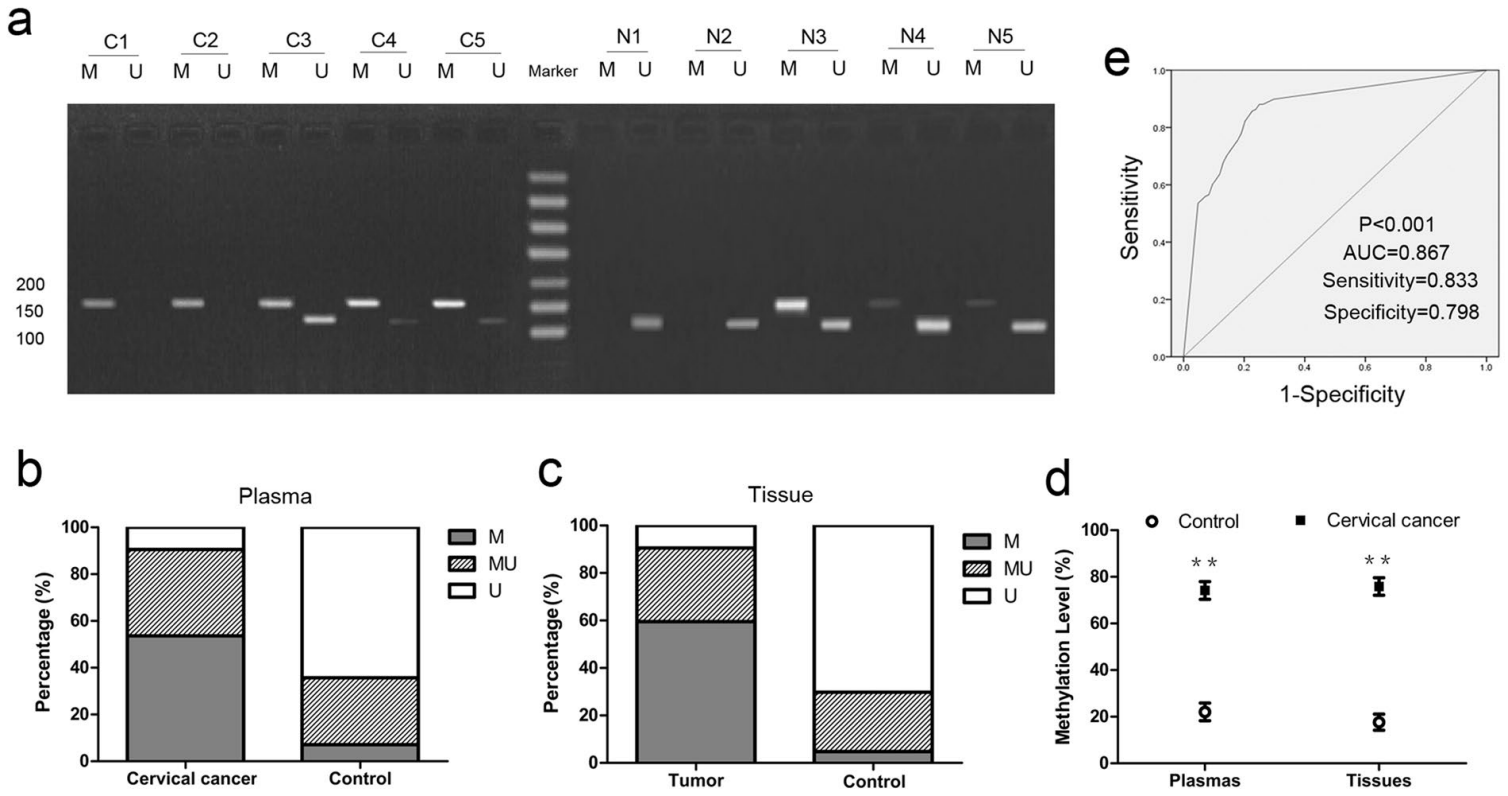
Analysis of methylation status of MEG3 promoter in plasmas and tissues using MSP. (**a**) MSP products illustrated that methylated pattern was largely detected in plasmas of cervical cancer patients (C1-C5 as examples) while unmethylated pattern was found in most of healthy participants’ plasmas (N1–N5 as examples). (**b**) The percentage of M, MU and U in plasmas of cervical cancer patients and healthy participants respectively. (**c**) The percentage of M, MU and U in cervical cancer tissues and adjacent normal tissues respectively. (**d**) The methylation level of MEG3 promoter in cervical cancer patients’ plasmas and cervical cancer tissues was significantly higher compared with their controls respectively. (**e**) ROC curve analysis for the diagnosis of cervical cancer by MEG3 methylation in plasma. MSP, methylation-specific polymerase chain reaction; M, methylated pattern; MU, partially methylated pattern; U, unmethylated pattern. **P < 0.01.

**Figure 2:**
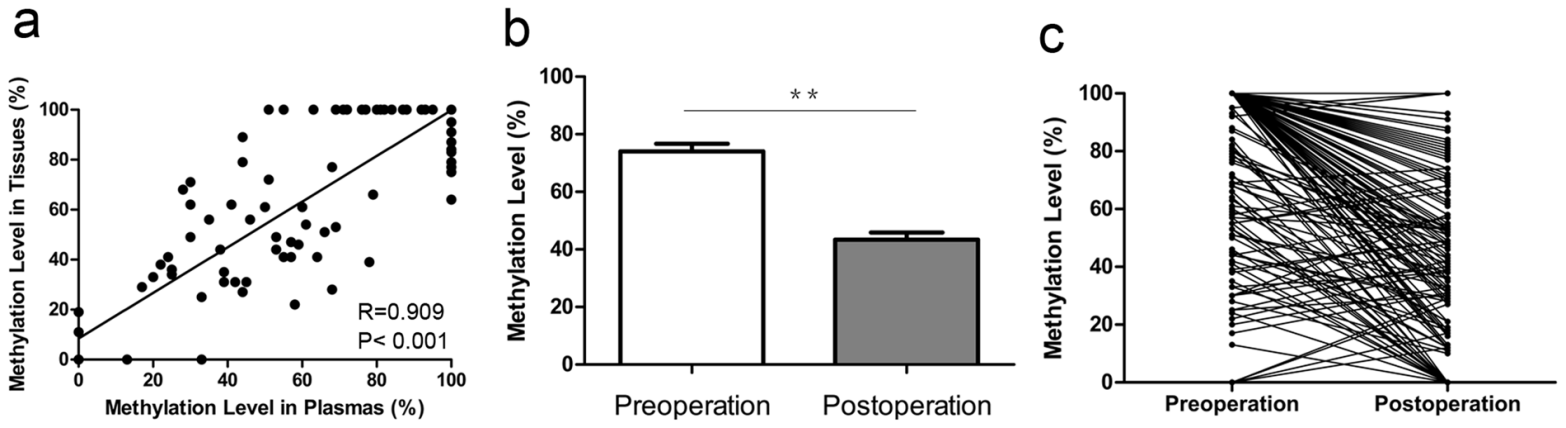
The reliability of MEG3 promoter methylation in plasma. (**a**) The level of MEG3 methylation in plasma presented a notable positive correlation with that in tissues of same patient. (**b**,**c**) A significantly higher methylation level was observed in DNA samples from preoperative plasmas as compared to postoperative plasmas. ^**^P < 0.01.

The aberrant methylated pattern (M) of MEG3 promoter was observed in 53.6% (90 of 168) of plasma samples of cervical cancer patients. Meanwhile, the unmethylated pattern (U) was observed in 16 of the 168 (9.5%) plasma samples of cervical cancer patients (Fig. 1a,b). In comparison, 64.3% (108 of 168) of the plasma samples of healthy participants displayed an unmethylated pattern (U) (Fig. 1a,b). The statistical analysis showed that plasmas of cervical cancer patients expressed a significantly higher level of MEG3 methylation than that of healthy participants (Fig. 1d, Table 1). The area under the ROC curve (AUC) was 0.867 according to level of MEG3 methylation in plasma for the diagnosis of cervical cancer. The best point for diagnosis by level of MEG3 methylation in plasma was 37.5%, with a sensitivity of 0.833 and specificity of 0.798 (Fig. 1e). Meanwhile, we also obtained a significantly higher methylation level in DNA samples from cervical cancer tissues as compared with that from adjacent normal tissues (Fig. 1c,d, Table 1). Furthermore, a significant positive correlation between MEG3 methylation in plasmas and tissues was confirmed with Pearson correlation in the same patient (P < 0.001, R = 0.909, Fig. 2a). More important, we observed a decrease in methylation level of MEG3 promoter after operation in plasmas of cervical cancer patients (P < 0.001, Fig. 2b,c).

**Table 1:** The methylation status of MEG3 promoter in CIN III and cervical cancer patients.

	Methylation status		χ^2^	P	R	OR^a^ (95% CI)
	M, MU	U				
Plasmas						
CIN III	68 (89.5%)	8 (10.5%)	41.5	<0.001	0.522	13.033 (5.488–30.954)
Control	30 (39.5%)	46 (60.5%)				
Cervical cancer	152 (90.5%)	16 (9.5%)	108.2	<0.001	0.567	17.100 (9.346–31.287)
Normal	60 (35.7%)	108 (64.3%)				
Tissues						
Cervical cancer	152 (90.5%)	16 (9.5%)	129.1	<0.001	0.620	22.420 (12.156–41.352)
Normal	50 (29.8%)	118 (70.2%)				

^a^OR (Odds ratio).

### The relevance of MEG3 methylation and clinical pathological characteristics of cervical cancer patients

**Figure 3:**
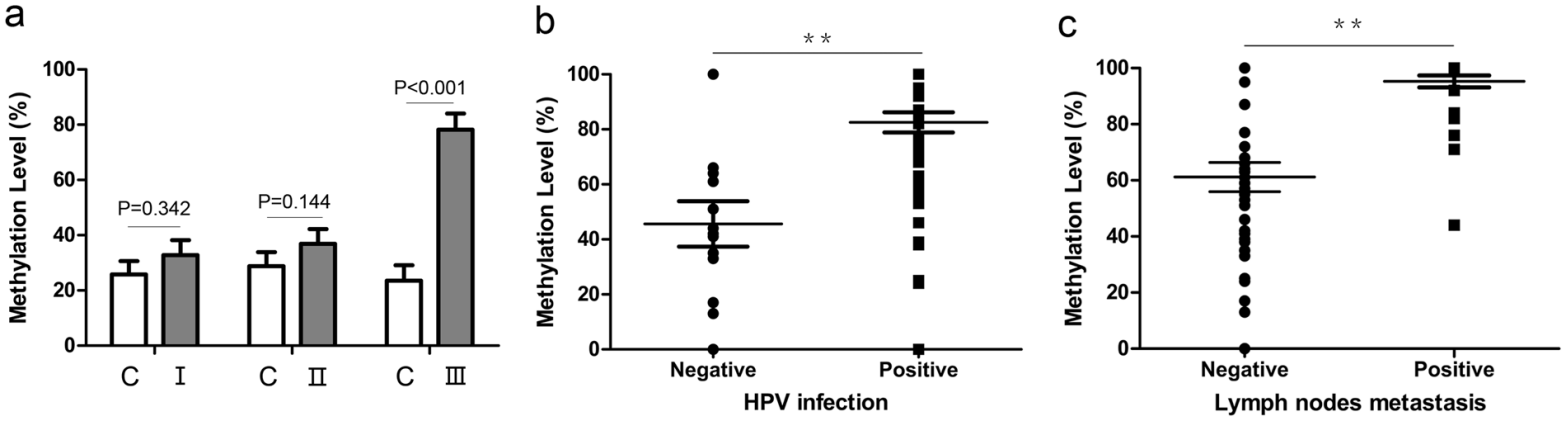
The correlation between MEG3 promoter methylation and clinical pathological characteristics of cervical cancer patients. (**a**) The methylation level of MEG3 promoter in CIN I (I), CIN II (II) and CIN III (III) patients’ plasmas. The methylation level of MEG3 promoter was significantly higher in HR-HPV positive (**b**), and lymph node metastasis (**c**) patients’ plasmas, compared with their controls respectively. C, control. ^**^P < 0.01.

There were no significant differences in plasma MEG3 methylation between CIN I–II and their matched healthy controls (Fig. 3a). But the level of MEG3 methylation was increased in plasmas of CIN III patients compared with that of healthy controls (P < 0.001, Fig. 3a). As shown in Fig. 3b, the methylation level of MEG3 in the plasmas was found to higher in HR-HPV positive patients compared with that in negative patients (P < 0.001). Moreover, the methylation level of MEG3 in the plasmas of cervical cancer patients with lymph node metastasis was significantly higher compared with those without metastasis (P < 0.001, Fig. 3c). In addition, Chi-square test also showed that hypermethylation of MEG3 promoter in plasma was a risk factor not only for CIN III and cervical cancer, but also HPV infection and lymph node metastasis (Tables 1 and 2). However, no significant associations were found between MEG3 methylation and age, menopause, histology, depth of invasion, differentiation, tumor size or Lymphatic vascular space invasion (LVSI) (Table 2).

**Table 2:** Relationship between MEG3 methylation in plasma and clinical pathological characteristics.

Clinical pathological characteristics		Methylation		P	R	OR^a^ (95% CI)
		M	MU, U			
Lymph nodes metastasis	Positive	52	12	<0.001	0.435	7.526 (3.577–15.835)
	Negative	38	66			
HPV infection	Positive	82	48	<0.001	0.353	6.406 (2.718–15.099)
	Negative	8	30			
Tumor size	≥4 cm	46	30	0.100		
	<4 cm	44	48			
Differentiation	Poorly	34	30	0.927		
	Well and Moderately	56	48			
Depth of invasion	>2/3	52	36	0.132		
	≤2/3	28	42			
LVSI	Positive	50	34	0.122		
	Negative	40	44			
Age	>50	42	40	0.551		
	≤50	48	38			
Menopause	Yes	30	36	0.090		
	No	60	42			
Histology	Squamous	76	60	0.216		
	Adenocarci-noma	14	18			

^a^OR (Odds ratio).

### Diagnostic value of MEG3 methylation as a plasma-based biomarker

**Figure 4:**
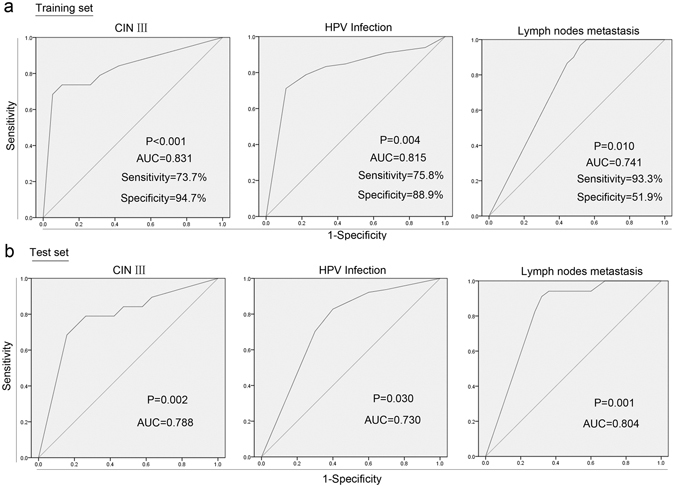
ROC curves for evaluating the diagnostic value of MEG3 methylation in plasmas. CIN III (included their match healthy participants) and cervical cancer patients were divided into training set and test set randomly and averagely. Both values of training set (**a**) and test set (**b**) fell in the fair to good range, thus presenting an well diagnostic power to premalignant lesion (CIN III), HR-HPV infection and lymph node metastasis.

First, we divided CIN III (include their match healthy participants) and cervical cancer patients into training set and test set randomly and averagely. Training set was used to set up model while test set for verification. Then we tested the diagnostic value of MEG3 methylation of plasma using ROC curve. In training set, the areas under the ROC curve (AUC) were 0.831, 0.815 and 0.741 for CIN III, HPV infection and lymph node metastasis, respectively (Fig. 4a). The best cutoffs were 52.0% (sensitivity: 73.7%; specificity: 94.7%), 70.5% (sensitivity: 75.8%; specificity: 88.9%) and 89.5% (sensitivity: 93.3%; specificity: 51.9%) for CIN III, HPV infection and lymph node metastasis, respectively (Fig. 4a).

In test set, AUCs were 0.788, 0.730 and 0.804 according to MEG3 methylation of plasma for the diagnosis of CIN III, HPV infection and lymph node metastasis respectively. When using best point of training set, the sensitivities were 84.2%, 78.1% and 82.4%, meanwhile specificities were 52.6%, 70.0% and 72.0% for CIN III, HPV infection and lymph node metastasis, respectively (Fig. 4b).

Moreover, we also calculated the sensitivity and specificity using formula method. The sensitivity was 81.3% and specificity was 63.5% for prediction of lymph node metastasis, while sensitivity and specificity was 63.1% and 84.2% for diagnosis of HPV infection (Table 3, n = 168). Then we evaluated the sensitivity and false negative rate of fluid based thin-layer cytological test (TCT) and MEG3 methylation for CIN III. The date showed that the sensitivity of TCT and MEG3 methylation was 87.5% and 79.2%, while the false negative rate of TCT and MEG3 methylation was 12.5% and 20.8% respectively (Table 4, n = 76). When combined with TCT and MEG3 methylation, the sensitivity and false negative rate was 93.1% and 6.9% respectively (Table 5, n = 76).

**Table 3:** Diagnostic value of MEG3 methylation (n = 168).

	Lymph nodes metastasis	HPV infection
Sensitivity	81.3%	63.1%
Specificity	63.5%	84.2%

**Table 4:** The sensitivity and False negative rate for CIN III (n = 76).

	TCT	Methylation	TCT + Methylation
Sensitivity	87.5%	79.2%	93.1%
False negative rate	12.5%	20.8%	6.9%

**Table 5:** Univariate and Multivariate analyses for recurrence-free survival.

Risk factors	Univariate analysis			Multivariate analysis		
	HR^a^	P value	95% CI	HR	P value	95% CI
MEG3 methylation	3.073	0.017	1.219~7.746	2.619	0.045	0.979~7.005
FIGO stage, (I, II)	2.405	0.032	1.078~5.362	3.124	0.015	1.249~7.813
Lymph nodes metastasis (Negative, Positive)	3.243	0.005	1.418~7.419	5.664	<0.0001	2.283~14.052
Differentiation (Well/Moderately, Poorly)	1.581	0.264	0.708~3.532			
Depth of invasion (≤2/3, >2/3)	1.124	0.775	0.503~2.510			
HR HPV infection (Negative, Positive)	2.404	0.155	0.717~8.064			
Tumor size	1.263	0.167	0.907~1.758			
LVSI (Negative, Positive)	0.690	0.371	0.307~1.554			
Age	1.003	0.863	0.967~1.040			
Histology (Squamous, Adenocarcinoma)	1.867	0.312	0.557~6.260			
Menopause (Yes, No)	1.444	0.370	0.647~3.225			

^a^HR (hazard ratio).

### Association of MEG3 methylation with prognosis of cervical cancer

**Figure 5:**
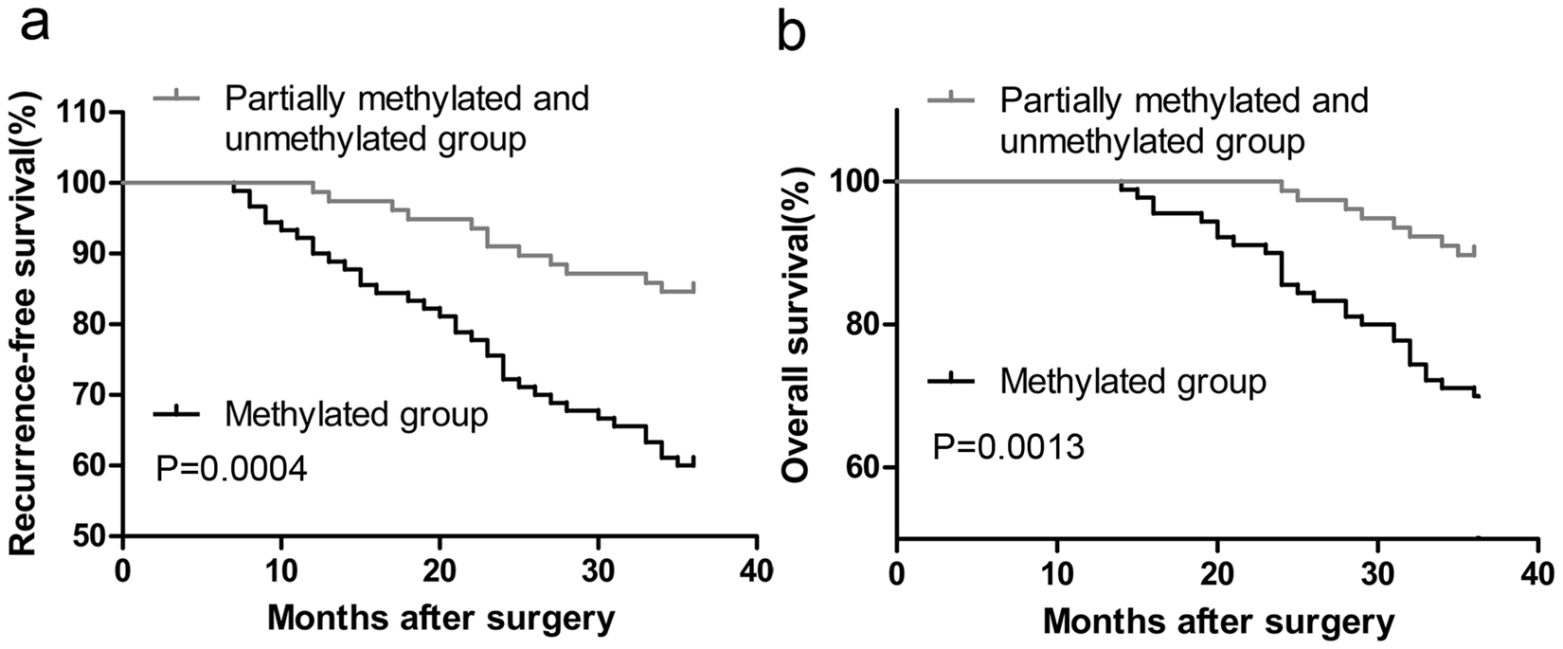
Association of MEG3 methylation with prognosis of cervical cancer patients. Kaplan-Meier curves predict the recurrence-free survival (**a**) and overall survival (**b**) according to the methylation status of MEG3 promoter in plasmas. P-values from log-rank test.

With regard to combined level of MEG3 methylation in plasma and prognosis, we divided the cervical cancer patients into 2 groups: group 1, methylated group (n = 90); group 2, partially methylated (M and U) and unmethylated group (n = 78). The Kaplan-Meier plot showed that patients with high level of MEG3 methylation had poorer recurrence-free survival (RFS) than those with low level of MEG3 methylation (P = 0.0004, Fig. 5a). On the other hand, the aberrant hypermethylation of MEG3 in plasma was also significantly associated with poorer overall survival (P = 0.0013, Fig. 5b).

Furthermore, univariate analysis of RFS revealed that the level of MEG3 methylation in plasma was a prognostic indicator for cervical cancer patients (Table 5). More important, Multivariate survival analysis further demonstrated that hypermethylation of MEG3 in plasma was an independent prognostic marker indicating poorer RFS for cervical cancer patients (Table 5).

## Discussion

Alternation of methylation pattern in cancer cell genomes is event taking place during the early stages of carcinogenesis and leading to profound modifications in gene expression^13^. Aberrant DNA methylation, which is the most common epigenetic change in human malignancies, may be used as a diagnosis, surveillance and prognostic biomarker according to recent studies^14–16^. Although plasma DNA methylation has been proved to be a potential biomarker for some diseases, the study forced on DNA methylation of cancer patients’ plasma is still limited^17, 18^.

The promoter region of MEG3 is rich in CpG dinucleotides. There are two differentially methylated regions (DMRs) located upstream of the MEG3 gene, IG-DMR and MEG3-DMR, which control the expression of MEG3^19^. It has been well proved that the loss of MEG3 expression in several types of human cancers was at least in part the result of hypermethylation in the MEG3 promoter region^20, 21^.

In our previous study^12^, we demonstrated that MEG3 was down-regulated in cervical cancer tissues. Moreover, MEG3 expression was negatively related with HR-HPV infection, FIGO stages and lymphatic metastasis. At last, we revealed that MEG3 functioned as a tumor suppressor by regulating miR-21-5p. However, the diagnostic value of plasma MEG3 methylation for cervical cancer patients is still unknown.

Based on others’ and our previous study, present study was designed to fill in this research gap. We first provided evidences to support that MEG3 promoter was hypermethylation not only in cervical cancer tissues, but also in plasmas of cervical cancer patients. We also observed that the level of MEG3 methylation in plasma was lower than that in cervical cancer tissues. DNA in plasma contains a larger background of DNA from normal cells may be one of the primary causes of it. Furthermore, we confirmed that the level of MEG3 methylation was significantly decreased in postoperative plasma, implying that MEG3 hypermethylation in plasma might be a result of the tumor tissue. More important, we found that the level of MEG3 methylation in plasma was positive related to that in tissue of same patient, indicating that the detection of MEG3 methylation level in plasma could in stead of that in tissue which made it more convenient and noninvasive. In summary, we provided strong evidences supporting that plasma MEG3 methylation could be a candidate of biomarker for cervical cancer.

Next, we focused on the diagnostic power of plasma MEG3 methylation. We first confirmed that hypermethylation of MEG3 in plasma was a risk factor for not only CIN III, but also HR-HPV infection and lymph node metastasis. That was consistent with the results of our previous study in tissues. Base on that, we further evaluated the diagnostic power of plasma MEG3 methylation for these relative factors. To make the results more credible, we divided patients and their match healthy participants into training set and test set. ROC curves indicated that plasma MEG3 methylation had high discriminating power to predict CIN III, HPV infection and lymph node metastasis in training set. Then we tested the optimal cutoff points in test set, showing that the sensitivity and specificity were still acceptable which made our results more credible. More important, we proved that the combination of TCT and MEG3 methylation was a much more powerful discrimination tool in predicting CIN III than TCT alone. For CIN III is a premalignant lesion of cervical cancer, our result implied that plasma MEG3 methylation might have great value in early diagnosis of cervical cancer. In addition, lymph node metastasis is a key factor in treatment decisions, suggesting that plasma MEG3 methylation might use for screening high-risk patients before therapy. Moreover, traditional tumor markers in plasma were almost the products of oncogene. The study focused on tumor suppressor gene as a plasma-based biomarker was still rare. In present study, we opened up a new perspective for exploration of plasma-based biomarker by analysis of DNA methylation of tumor suppressor gene.

For plasma MEG3 methylation was closely linked to lymph node metastasis of cervical cancer, we speculated that it might be associated with prognosis too. As anticipated, there was a trend toward the poorer RFS and overall survival in the patients with high level of MEG3 methylation in plasma. More important, Cox multivariate analysis suggested that plasma MEG3 methylation represented an independent prognostic factor for RFS. Thus, plasma MEG3 methylation may be useful as a prognostic marker for the prediction of relapse and survival in cervical cancer patients.

However, there is still much work should be done. Some techniques, such as methyl-BEAMing and Pyrosequencing, demonstrated higher efficiency and more sensitive than MSP recently^22^. In future studies, we will use these techniques to further confirm our data and make our results more accurate.

In conclusion, plasma MEG3 methylation may be a reliable diagnostic biomarker for the early stage, HR-HPV infection and lymph node metastasis of cervical cancer. Our study also supports its application as a prognostic biomarker for cervical cancer. Therefore, the methylation status of MEG3 in plasma is a potential novel epigenetic biomarker which has great value in the clinical practice of cervical cancer.

## Materials and Methods

### Human samples

Plasma samples were collected from 36 CIN I, 48 CIN II, 76 CIN III and 168 cervical cancer patients between July 2010 and December 2012. And plasma samples from 328 matched healthy participants were collected as controls. The tumor tissues and adjacent normal tissues were obtained from 168 cervical cancer patients who underwent primary surgery and did not receive any pre-operative cancer treatments. All of the CIN patients has received ThinPrep cytology test (TCT) and colposcopic biopsy, and final diagnosis was made according to the histological diagnosis of tissues after biopsy. All samples were confirmed by at least 2 pathologists. All tissue samples were immediately frozen in liquid nitrogen following resection and stored at −80 °C until use. Plasma collection time points of cervical cancer patients were included preoperation (before surgery) and postoperation (a month after surgery). Peripheral blood samples were centrifuged at 2000 rpm for 15 min at 4 °C immediately and the supernatants were then stored at −80 °C. Informed consent was obtained from all subjects and the study was approved by the Ethics Committee of Shenzhen People’s Hospital. All clinical investigations were conducted in accordance with the principles of the Declaration of Helsinki. Detailed patients characteristics are listed in Table 2.

### Methylation assays

Genomic DNA from plasmas and tissues was first extracted using QIAamp blood mini kit (Qiagen, German) and QIAmp DNA Mini kit (Qiagen) respectively, according to product manual. The OD260/OD280 values of DNA samples were between 1.7 and 1.9. And the concentration of the DNA samples was 10 ng/ul. Then the bisulfite conversion of Genomic DNA was performed using EpiTect Bisulfite kit (Qiagen) following the manufacturer’s protocol. The methylation statuses of MEG3 promoter were evaluated by methylation-specific polymerase chain reaction (MSP) using EpiTect MSP kit (Qiagen). PCR amplification was performed using the following cycling conditions: 95 °C 15 min; 94 °C 30 s, 70 °C 30 s, 72 °C 30 s, 5 cycles; 94 °C 30 s, 65 °C 30 s, 72 °C 30 s, 5 cycles; 94 °C 30 s, 60 °C 30 s, 72 °C 30 s, 30 cycles; 72 °C 7 min. The PCR products were separated on a 2.5% agarose gel, stained with ethidium bromide, and visualized under UV illumination. Finally, the gray scanning was carried out by Quantity One software (Bio-Rad, California, US). The primers for the methylated (M) reaction were as follows: 5′-GTT AGT AAT CGG GTT TGT CGG C (sense) and 5′-AAT CAT AAC TCC GAA CAC CCG CG (antisense), with a 160-bp amplification product. The primers for the unmethylated (U) reaction were as follows: 5′-GAG GAT GGT TAG TTA TTG GGG T (sense) and 5′-CCA CCA TAA CCA ACA CCC TAT AAT CAC A (antisense), with a 120-bp amplification product. The percentage of methylation was calculated using following method: gray value of methylation/(gray value of methylation + gray value of unmethylation).

### Statistical analysis

All experiments were repeated independently three times and data was presented as the mean ± standard deviation. Associations between MEG3 methylation and clinical-pathologic characteristics were analyzed by the chi-square test. Receiver operating characteristic (ROC) curves were used to determine the diagnostic value for the patients in this study. Survival probability was analyzed by the Kaplan-Meier methods and evaluated by log-rank test. Cox regression model was also performed to assess the associations of MEG3 methylation with disease relapse or death. Comparisons between groups were evaluated by Student’s t test or LSD test using SPSS 11.0 software (SPSS, Chicago, IL, USA). P < 0.05 was regarded as statistically significant.
